# Human Papillomavirus Knowledge, Attitudes, and Vaccination-Related Practices Among Medical Students in Himachal Pradesh, India: A Multicenter Analysis

**DOI:** 10.7759/cureus.114382

**Published:** 2026-08-11

**Authors:** Shardul S Rathore, Sandeep S Rathore, Monia Rathore, Kavita Mardi, Atul Sharma

**Affiliations:** 1 Department of Obstetrics and Gynecology, Indira Gandhi Medical College, Shimla, IND; 2 Department of Physiology, Indira Gandhi Medical College, Shimla, IND; 3 Department of Pathology, Indira Gandhi Medical College, Shimla, IND

**Keywords:** attitudes, hpv vaccination, human papillomavirus, knowledge, medical students, practices, vaccine uptake

## Abstract

Background: Medical students will play an important role in counseling patients about human papillomavirus (HPV) vaccination. Evidence on the gap between HPV awareness, detailed knowledge, and actual vaccine uptake among future healthcare providers is particularly relevant as HPV vaccination is incorporated into India’s nationwide vaccination program.

Methods: A multicenter cross-sectional survey was conducted from February to July 2026 across seven medical colleges. Participating students completed an expert-reviewed and pilot-tested 52-item online questionnaire. Knowledge scores ranged from 0 to 15, and attitude scores ranged from 8 to 40, while practice-related items were analyzed individually. Knowledge and attitude scores were compared across participant characteristics; their relationship was assessed using Pearson’s correlation coefficient, and factors independently associated with vaccination uptake were examined using multivariable binary logistic regression.

Results: Among 1,159 participants, 1,078 (93.0%) had heard of HPV, but only 134 (11.6%) had been vaccinated. The mean knowledge score was 7.77 ± 3.63; 483 (41.7%) participants had lower knowledge and 181 (15.6%) had higher knowledge. The mean attitude score was 30.8 ± 4.58, with 853 (73.6%) demonstrating a favorable attitude. Knowledge and attitude scores were moderately correlated (r = 0.418, p < 0.001). The leading reasons for nonvaccination among the 1,025 unvaccinated were the absence of a recommendation, reported by 288 (28.1%), and lack of awareness, reported by 287 (28.0%). In the adjusted analysis, vaccination uptake was associated with a higher knowledge score (adjusted odds ratio (aOR) per point: 1.073, 95% confidence interval (CI): 1.009-1.141; p = 0.023), female gender (aOR: 1.748, 95% CI: 1.149-2.658; p = 0.007), and urban residence (aOR: 2.212, 95% CI: 1.488-3.290; p < 0.001). Attitude score and study phase were not independently associated with vaccination uptake.

Conclusion: Despite high awareness and generally favorable attitudes, detailed HPV knowledge and vaccination uptake remained low. Medical colleges should strengthen practical, gender-neutral HPV education and combine counseling with accessible and affordable vaccination services.

## Introduction

Human papillomavirus (HPV) is one of the most common sexually transmitted viral infections worldwide and is responsible for a considerable burden of preventable disease [1]. Among the diseases associated with HPV, cervical cancer remains a major public health concern. The burden is particularly high in low- and middle-income countries, where access to vaccination, screening, and regular surveillance is often limited. Global Cancer Observatory 2024 estimated approximately 604,000 new cases of cervical cancer and 280,000 deaths globally. Cervical cancer was the fifth most commonly diagnosed cancer among women and the fourth leading cause of cancer death among women [2]. India accounted for 127,526 new cases in 2022 and nearly one-fourth of cervical cancer deaths worldwide [3]. These figures remain concerning because cervical cancer is largely preventable.

More than 200 HPV genotypes have been identified. HPV types 16 and 18 are responsible for most cases of cervical cancer. Other high-risk types, including 31, 33, 45, 52, and 58, play an important role in carcinogenesis. Persistent infection with a high-risk HPV type is the main etiological factor in the development of cervical intraepithelial neoplasia and invasive cervical carcinoma. Low-risk HPV types, particularly 6 and 11, are more commonly associated with benign lesions such as anogenital warts. HPV infection is not limited to cervical disease. It is also associated with anal, vulvar, vaginal, penile, and oropharyngeal cancers and therefore affects both women and men [4].

HPV vaccination is most effective when given before exposure to the virus. It provides protection against the HPV types responsible for most HPV-related cancers and precancerous lesions. The World Health Organization has included HPV vaccination as a key component of its strategy for the elimination of cervical cancer and recommends routine vaccination of girls aged 9-14 years, preferably before the onset of sexual activity. Countries with high vaccination coverage have reported reductions in HPV infection, genital warts, and high-grade cervical lesions [5].

In India, bivalent and quadrivalent HPV vaccines were licensed for prescription use in 2008 and subsequently became available through the private sector. The introduction of CERVAVAC (Serum Institute of India Pvt. Ltd., Pune, India), India’s first indigenously developed quadrivalent HPV vaccine in 2023, added a domestically manufactured vaccine option [6]. However, vaccine uptake remained low for many years. Cost, inadequate awareness, safety concerns, and the lack of a national immunization program were among the commonly reported barriers [7].

The launch of India’s nationwide HPV vaccination program on February 28, 2026, marked the introduction of HPV vaccination into the country’s national immunization program. The program provides a single dose of the quadrivalent HPV vaccine, Gardasil-4, free of cost to approximately 11.5 million girls aged 14 years across all states and union territories. Following the initial three-month campaign, the vaccine will continue to be available on routine immunization days [8].

Medical students form an important group in this context. Their knowledge and attitudes during training may later influence how they counsel patients and families. As future doctors, they are also likely to be asked questions regarding vaccine safety, eligibility, dosing, and the vaccination of boys and men. Adequate knowledge during undergraduate training may therefore improve vaccine counseling and help address common misconceptions [9].

The undergraduate MBBS program in India comprises four and a half academic years followed by a one-year compulsory rotating medical internship [10]. The academic program is organized into Phase I, Phase II, Phase III Part I, and Phase III Part II. Although clinical integration and early clinical exposure begin during Phase I, the extent of clinical training increases progressively in the later phases. Consequently, students at different stages of the program have varying levels of academic and clinical exposure, which may influence their knowledge and perceptions regarding HPV infection and vaccination.

Studies among medical students in different countries have generally shown good overall awareness of HPV and cervical cancer. However, detailed knowledge is often incomplete. Gaps have been reported regarding vaccine indications, recommended schedules, protection against noncervical cancers, and vaccination of men [11]. Indian studies have shown a similar pattern. Although many medical students have heard of HPV and its vaccine, actual vaccination rates remain low. Cost, fear of adverse effects, lack of sufficient information, and social beliefs related to sexually transmitted infections have been reported as barriers to vaccination [12,13].

Evidence from Himachal Pradesh is limited. Previous research has included a single-center study among medical and nursing students and staff nurses as well as a community-based assessment among adults in the state [14,15]. To our knowledge, no large multi-institutional study has assessed HPV-related knowledge, attitudes, and practices (KAPs) among MBBS students across the medical colleges of the state. Baseline information from this group is especially relevant now that HPV vaccination is being introduced through the national immunization program and healthcare professionals will eventually be involved in counseling and vaccine promotion [8].

To our knowledge, this is the first multicenter study across Himachal Pradesh to assess HPV-related KAPs among MBBS students. The study aimed to quantify the gap between prior HPV awareness, detailed HPV-related knowledge, and actual vaccination uptake among MBBS students across seven medical colleges in Himachal Pradesh. It also examined demographic and academic factors independently associated with vaccination uptake and identified educational, social, and service-related barriers that may inform interventions within medical colleges.

## Materials and methods

Study design, setting, and duration

This multicenter, cross-sectional, questionnaire-based KAP study was conducted among undergraduate medical students in Himachal Pradesh, India. The study was conducted by the Department of Obstetrics and Gynecology, Indira Gandhi Medical College (IGMC), Shimla.

The participating institutions comprised six state government medical colleges and one private medical college in Himachal Pradesh: IGMC, Shimla; Dr Rajendra Prasad Government Medical College, Tanda; Shri Lal Bahadur Shastri Government Medical College, Mandi; Dr Yashwant Singh Parmar Government Medical College, Nahan; Dr Radhakrishnan Government Medical College, Hamirpur; Pandit Jawaharlal Nehru Government Medical College, Chamba; and Maharishi Markandeshwar Medical College, Solan. All India Institute of Medical Sciences, Bilaspur, a centrally administered medical institute located in Himachal Pradesh, was not included in the study.

The study was conducted from February to July 2026, including questionnaire finalization, pilot testing, data collection, and preliminary data analysis. Questionnaire responses were collected between May and June 2026.

Study population, eligibility, and recruitment

The study population comprised MBBS students in Phase I, Phase II, Phase III Part I, Phase III Part II, or internship at the seven participating institutions. Thus, a total-population invitation or attempted census approach was adopted rather than selecting a sample from the eligible population. Students were included if they were currently enrolled in one of the participating institutions, provided electronic informed consent, and submitted the questionnaire. Students who did not provide consent were excluded.

The minimum required sample size was calculated using Cochran’s formula for estimating a population proportion [16]. Assuming a 95% confidence level, a 5% absolute margin of error, and an expected proportion of 50%, the initial sample size was calculated as 385 participants. After applying the finite population correction for an estimated eligible population of 4,350 MBBS students, the minimum required sample size was 354 participants. After allowing for an anticipated nonparticipation proportion of 10%, the final target sample size was increased to 394 participants. However, rather than restricting recruitment to the calculated minimum, all eligible students from the participating institutions were invited to participate.

Questionnaire development and content validation

The questionnaire was developed by the principal investigator following a review of the published literature on KAPs related to HPV infection, HPV vaccination, and cervical cancer among medical and other health-professional students [12,13]. The questionnaire was newly designed and worded (see the Appendix).

The initial questionnaire was reviewed for face and content reliability by three subject experts from the departments of Obstetrics and Gynecology, Community Medicine, and Pathology. The expert panel assessed the relevance, clarity, comprehensibility, and coverage of the questionnaire items. Their recommendations were incorporated into the final version of the instrument.

Pilot testing and reliability assessment

Before full-scale administration, the questionnaire was pilot-tested among 21 respondents to assess its clarity and the internal consistency of the knowledge and attitude domains. Cronbach’s alpha was calculated separately for the two domains using the pilot-test responses. The 15-item knowledge domain demonstrated a Cronbach’s alpha of 0.834, whereas the eight-item attitude domain demonstrated a Cronbach’s alpha of 0.723. Pilot-test participants were excluded from the main analysis.

Questionnaire structure

The final questionnaire contained 52 items organized into six sections: 1) sociodemographic and academic characteristics and prior awareness of HPV: six items; 2) knowledge regarding HPV infection and HPV vaccination: 15 items; 3) attitudes toward HPV infection and vaccination: eight items; 4) vaccination practices, vaccination-related behaviors, and educational preferences: eight items; 5) social, cultural, and religious beliefs and misconceptions regarding HPV and HPV vaccination: 13 items; and 6) sources of information and reasons for nonvaccination: two items.

The knowledge section assessed HPV transmission, associated diseases, vaccination schedules, target groups, contraindications, and postvaccination screening. The attitude section assessed perceived seriousness, vaccine safety and effectiveness, and willingness to recommend vaccination. The practice section assessed vaccination status and related behaviors. The social beliefs and misconceptions section assessed common beliefs and myths regarding vaccination.

Scoring methodology

For the knowledge domain, each correct response was awarded 1 point, whereas an incorrect or “I don’t know” response was awarded 0 points. For multiple-response knowledge items, a response was classified as correct only when all correct options and no incorrect options were selected. The total knowledge score ranged from 0 to 15. Knowledge scores were categorized as lower (0-7), intermediate (8-11), and higher (12-15).

Attitude items were rated using a five-point Likert scale ranging from “strongly disagree” to “strongly agree.” Positively worded items were scored in ascending order, whereas one negatively worded item was reverse-scored so that higher total scores consistently represented a more favorable attitude toward HPV vaccination. The total attitude score ranged from 8 to 40. Attitude scores were categorized a priori as unfavorable (<21), neutral (21-28), and favorable (≥29).

Practice items were analyzed individually and were not combined into a composite practice score. Items assessing social beliefs, misconceptions, sources of information, and educational needs were also summarized individually.

Questionnaire administration and data collection

The final questionnaire was administered electronically using Google Forms (Google LLC, Mountain View, CA). An online format was selected to facilitate standardized data collection across geographically dispersed medical colleges.

Junior residents from the departments of Obstetrics and Gynecology at the participating institutions were requested to visit undergraduate teaching sessions, briefly explain the purpose of the study, and invite eligible students to participate. The questionnaire link or QR code was then shared with the students, who completed the survey voluntarily using their personal electronic devices. Sign-in was required to limit each participant to a single response; email addresses were not recorded to maintain anonymity. No financial or academic incentives were offered for participation.

The questionnaire was configured so that all study items were mandatory after consent was provided, except the optional multiple-response item assessing reasons for nonvaccination, which was presented to participants who reported that they had not received the HPV vaccine. The number of participants who answered this optional item was recorded and used as the denominator when calculating percentages for reasons for nonvaccination. Consequently, no partially completed submitted questionnaires were recorded for the mandatory items.

Ethical considerations

Ethical approval was obtained from the Institutional Ethics Committee of IGMC, Shimla (approval no: HFW/MC/IEC//2026-4, dated March 28, 2026).

An electronic participant information sheet was displayed at the beginning of the questionnaire. It described the purpose of the study, the voluntary nature of participation, the study procedures, and the measures used to protect confidentiality. Electronic informed consent was obtained before participants were permitted to proceed to the questionnaire.

Participation was voluntary, and respondents could withdraw at any stage before submitting the completed form. No personally identifying information was collected, and responses were analyzed anonymously.

Data management

Responses submitted through Google Forms were compiled into a spreadsheet and exported to Microsoft Excel (Microsoft Corporation, Redmond, WA) for coding and data management. The electronic dataset was password-protected and accessible only to the study investigators. The dataset was screened for duplicate submissions, coding errors, internal inconsistencies, and implausible values. Categorical variables were coded numerically, and positively and negatively worded attitude items were scored or reverse-scored according to the predefined scoring scheme. After data cleaning, the dataset was imported into jamovi version 2.7 (The jamovi project, Sydney, Australia) for statistical analysis.

Statistical analysis

Categorical variables were summarized using frequencies and percentages. Continuous variables were summarized using means and standard deviations when approximately normally distributed and medians with interquartile ranges (IQRs) when their distributions were skewed.

Internal consistency of the knowledge and attitude domains was also assessed in the full study sample using Cronbach’s alpha. Knowledge and attitude scores were compared across participant characteristics using independent-sample Student’s t-test when the assumption of homogeneity of variances was satisfied and Welch’s t-test when this assumption was violated. Comparisons involving more than two groups were performed using Welch’s one-way analysis of variance (ANOVA), followed by Games-Howell post hoc testing for pairwise comparisons. Associations between categorical variables, including HPV vaccination status and sociodemographic, academic, social, and belief-related characteristics, were examined using Pearson’s chi-square test. Fisher’s exact test was used when the assumptions of the chi-square test were not satisfied because of small expected cell frequencies. Cramer’s V was reported as a measure of effect size for selected chi-square associations. The relationship between knowledge and attitude scores was assessed using Pearson’s correlation coefficient.

Multivariable binary logistic regression analysis was conducted to identify factors independently associated with HPV vaccination uptake. The dependent variable was coded as having received the HPV vaccine (yes versus no). Knowledge and attitude scores were entered as continuous variables, while gender, phase of study, and place of residence were entered as categorical variables. Male gender, Phase I, and rural residence were used as the respective reference categories. All specified predictors were entered simultaneously into the multivariable model. Adjusted odds ratios (aORs) with 95% confidence intervals (CIs) were reported. The overall effect of phase of study was evaluated using an omnibus likelihood-ratio test. All statistical tests were two-sided, and a p value of <0.05 was considered statistically significant.

Measures to reduce bias

Several measures were incorporated to reduce potential bias. All eligible students across the participating institutions were invited to participate to broaden coverage of the target population. A standardized electronic questionnaire and uniform participant information were used across institutions. Anonymous data collection and the absence of academic or financial incentives were intended to reduce social desirability and response-related pressures. Mandatory questionnaire fields prevented missing item-level data among submitted responses. Nevertheless, differences in participation across institutions and professional years could have introduced nonresponse bias.

Reporting guideline

The study is reported in accordance with the Strengthening the Reporting of Observational Studies in Epidemiology guidelines for cross-sectional studies [17].

## Results

Of approximately 4,350 eligible MBBS students, 1,159 participated, corresponding to a participation proportion of 1,159/4,350 (26.6%). The mean age of the participants was 20.9 ± 1.65 years, as shown in Table 1. Female individuals constituted 715 (61.7%) of the sample, while male individuals constituted 444 (38.3%). Phase I students represented the largest group, with 503 (43.4%) participants, followed by Phase II students, with 219 (18.9%); Phase III Part II students, with 212 (18.3%); Phase III Part I students, with 177 (15.3%); and interns, with 48 (4.1%). Participants were recruited from seven medical colleges, with the largest proportion enrolled at IGMC Shimla, comprising 297 (25.6%). Overall, 644 (55.6%) of the participants were urban residents. Most participants had previously heard of HPV, with 1,078 (93.0%) reporting prior awareness, whereas 81 (7.0%) had not heard of HPV. Only 134 students (11.6%) had received the HPV vaccine, whereas 1,025 (88.4%) were unvaccinated.

**Table 1:** Demographic characteristics of study participants (n = 1,159) Data are presented as frequency (n) and percentage (%), unless otherwise specified. Age is presented as mean ± SD. This table contains descriptive statistics only; no inferential statistical test was applied SD: standard deviation; IGMC: Indira Gandhi Medical College; Dr RPGMC: Dr Rajendra Prasad Government Medical College; SLBSGMC: Shri Lal Bahadur Shastri Government Medical College; Pt JLNGMC: Pandit Jawaharlal Nehru Government Medical College; Dr YSPGMC: Dr Yashwant Singh Parmar Government Medical College; Dr RKGMC: Dr Radhakrishnan Government Medical College; HPV: human papillomavirus

Characteristic	Category	Value
Age (years), mean ± SD	-	20.9 ± 1.65
Gender, n (%)	Male	444 (38.3)
	Female	715 (61.7)
Phase of study, n (%)	Phase I	503 (43.4)
	Phase II	219 (18.9)
	Phase III Part I	177 (15.3)
	Phase III Part II	212 (18.3)
	Internship	48 (4.1)
Institution, n (%)	IGMC Shimla	297 (25.6)
	Dr RPGMC Tanda	158 (13.6)
	SLBSGMC Mandi	116 (10.0)
	Pt JLNGMC Chamba	155 (13.4)
	Dr YSPGMC Nahan	155 (13.4)
	Dr RKGMC Hamirpur	181 (15.6)
	MMMC Solan	97 (8.4)
Residence, n (%)	Rural	515 (44.4)
	Urban	644 (55.6)
Previously heard of HPV, n (%)	Yes	1,078 (93.0)
	No	81 (7.0)
Vaccinated, n (%)	Yes	134 (11.6)
	No	1,025 (88.4)

Knowledge was highest regarding the primary mode of HPV transmission, correctly identified by 919 (79.3%) participants; the association of high-risk HPV types with cancer, identified by 913 (78.8%); and the existence of multiple HPV types, identified by 891 (76.9%). In contrast, only 237 (20.4%) correctly identified all diseases associated with HPV, and 62 (5.3%) correctly identified the full range of conditions against which HPV vaccination provides protection. Knowledge was also limited regarding contraindications to HPV vaccination, correctly identified by 332 (28.6%), and the recommended number of vaccine doses for individuals older than 15 years, correctly identified by 431 (37.2%), as shown in Table 2.

**Table 2:** Knowledge regarding HPV infection and vaccination among study participants Data are presented as frequency (n) and percentage (%). For multiple-response knowledge items, a response was classified as correct only when all correct options and no incorrect options were selected. Incorrect and “don’t know” responses were combined. This table contains descriptive statistics only; no inferential statistical test was applied HPV: human papillomavirus

Knowledge item	Correct response, n (%)	Incorrect or “don’t know” response, n (%)
HPV can cause which of the following? (Select all that apply)	237 (20.4)	922 (79.6)
What is the primary mode of HPV transmission?	919 (79.3)	240 (20.7)
Is cervical cancer caused only by HPV?	733 (63.2)	426 (36.8)
Is there more than one type of HPV?	891 (76.9)	268 (23.1)
High-risk HPV types are primarily associated with:	913 (78.8)	246 (21.2)
At what age is HPV vaccination ideally recommended?	673 (58.1)	486 (41.9)
Does being vaccinated for HPV mean you can't get cervical cancer?	749 (64.6)	410 (35.4)
Should men also get vaccinated for HPV?	728 (62.8)	431 (37.2)
How many doses are recommended for individuals aged 9-14 years?	509 (43.9)	650 (56.1)
How many doses are recommended for individuals older than 15 years?	431 (37.2)	728 (62.8)
Is the HPV vaccine included in India's National Immunization Schedule?	461 (39.8)	698 (60.2)
Which of the following are contraindications to HPV vaccination? (Select all that apply)	332 (28.6)	827 (71.4)
At what frequency should cervical cancer screening be done in vaccinated women?	627 (54.1)	532 (45.9)
Is routine screening still required after HPV vaccination?	743 (64.1)	416 (35.9)
HPV vaccination provides protection against: (Select all that apply)	62 (5.3)	1,097 (94.7)

The mean knowledge score was 7.77 ± 3.63 out of 15, with a median of 8 and a range of 0-14, as seen in Table 3. Based on the predefined knowledge categories, 181 (15.6%) participants had higher knowledge, 495 (42.7%) had intermediate knowledge, and 483 (41.7%) had lower knowledge. The mean attitude score was 30.8 ± 4.58 out of 40, with a median of 31 and a range of 12-39. Based on the predefined attitude categories, 853 (73.6%) participants had a favorable attitude, 271 (23.4%) had a neutral attitude, and 35 (3.0%) had an unfavorable attitude toward HPV infection and vaccination. A moderate positive correlation was observed between knowledge and attitude scores (Pearson’s r = 0.418, p < 0.001), indicating that higher knowledge scores were associated with more favorable attitude scores. Cronbach’s alpha was 0.818 for the 15-item knowledge domain and 0.734 for the eight-item attitude domain, indicating good and acceptable internal consistency, respectively.

**Table 3:** Knowledge and attitude scores and category summary (n = 1,159) Data are presented as mean ± SD, median, range, and frequency (n) with percentage (%), as appropriate. The possible knowledge-score range was 0-15, and the possible attitude-score range was 8-40 SD: standard deviation

Domain	Measure	Category	Value
Knowledge	Mean	-	7.77
	Standard deviation	-	3.63
	Median	-	8
	Minimum	-	0
	Maximum	-	14
	Category distribution, n (%)	Higher	181 (15.6)
		Intermediate	495 (42.7)
		Lower	483 (41.7)
Attitude	Mean	-	30.8
	Standard deviation	-	4.58
	Median	-	31
	Minimum	-	12
	Maximum	-	39
	Category distribution, n (%)	Favorable	853 (73.6)
		Neutral	271 (23.4)
		Unfavorable	35 (3.0)

Most participants agreed or strongly agreed that HPV was a serious infection, 997 (86.0%), and that HPV vaccination should be mandatory for adolescent girls, 1,037 (89.5%), as shown in Table 4. Nearly two-thirds, 753 (65.0%), supported encouraging HPV vaccination among boys and men. Most participants also supported providing the vaccine free of cost to medical students, 946 (81.6%); stated that they would recommend vaccination to family members, 929 (80.2%); felt confident explaining its benefits to society, 899 (77.6%); and believed that HPV vaccination could reduce the incidence of cervical cancer in India, 957 (82.6%). Regarding adverse effects, 394 (34.0%) agreed or strongly agreed that they were worried about adverse effects, whereas 500 (43.1%) gave a neutral response.

**Table 4:** Attitude toward HPV infection and vaccination among study participants (n = 1,159) Responses are presented as frequency (n) and percentage (%) for each category of the five-point Likert scale. The negatively worded item concerning vaccine side effects was reverse-scored only when calculating the total attitude score; the original response distribution is presented in this table. The table contains descriptive statistics only HPV: human papillomavirus

Attitude statement	Strongly agree, n (%)	Agree, n (%)	Neutral, n (%)	Disagree, n (%)	Strongly disagree, n (%)
HPV is a serious infection	586 (50.6)	411 (35.5)	123 (10.6)	25 (2.1)	14 (1.2)
HPV vaccination should be mandatory for adolescent girls	663 (57.2)	374 (32.3)	86 (7.4)	14 (1.2)	22 (1.9)
HPV vaccination should be encouraged for boys/men as well	283 (24.4)	470 (40.6)	275 (23.7)	36 (3.1)	95 (8.2)
I worry about the side effects of the HPV vaccine	82 (7.1)	312 (26.9)	500 (43.1)	184 (15.9)	81 (7.0)
HPV vaccination should be provided free of cost to all medical students	598 (51.6)	348 (30.0)	126 (10.9)	23 (2.0)	64 (5.5)
I would recommend HPV vaccination to my family members	448 (38.7)	481 (41.5)	159 (13.7)	25 (2.2)	46 (4.0)
I feel confident explaining the benefits of HPV vaccination to society	376 (32.4)	523 (45.1)	185 (16.0)	31 (2.7)	44 (3.8)
I believe that HPV vaccination can reduce the incidence of cervical cancer in India	508 (43.8)	449 (38.7)	139 (12.0)	25 (2.2)	38 (3.3)

Knowledge and attitude scores varied across selected demographic characteristics; details are shown in Table 5. Female students had significantly higher mean knowledge scores than male students (8.50 ± 3.26 vs. 6.60 ± 3.88; Welch’s t(822) = -8.59, p < 0.001). Female students also had higher mean attitude scores than male students (31.4 ± 4.33 vs. 29.8 ± 4.79), and this difference was statistically significant (Welch’s t(868) = -5.83, p < 0.001). Knowledge scores differed significantly across phases of study (Welch’s ANOVA, F(4, 284) = 65.0, p < 0.001). A progressive increase in mean knowledge score was observed from Phase I students (6.13 ± 3.85) to interns (10.04 ± 2.15). Games-Howell post hoc comparisons showed that Phase I students had significantly lower knowledge scores than all other year groups (all p < 0.001). Phase II students had significantly lower scores than Phase III Part II students and interns (both p < 0.001) but did not differ significantly from Phase III Part I students (p = 0.266). Phase III Part I students had significantly lower scores than Phase III Part II students (p = 0.020) and interns (p = 0.017). The difference between Phase III Part II students and interns was not statistically significant (p = 0.835). Attitude scores also differed significantly according to phase of study (Welch’s ANOVA, F(4, 261) = 27.7, p < 0.001), with the highest mean score observed among Phase III Part II students (32.9 ± 4.05). Games-Howell post hoc comparisons showed that Phase I students had significantly lower attitude scores than Phase III Part I students (p < 0.001), Phase III Part II students (p < 0.001), and interns (p = 0.008). Phase II students also had significantly lower attitude scores than Phase III Part I students (p = 0.004) and Phase III Part II students (p < 0.001). The remaining pairwise comparisons were not statistically significant.

**Table 5:** Association of knowledge and attitude scores with participant characteristics Knowledge and attitude scores are presented as mean ± SD ^*^Welch’s independent-samples t-test ^†^Independent-samples Student’s t-test ^‡^Welch’s one-way ANOVA, followed by Games-Howell post hoc tests for pairwise comparisons All tests were two-sided, and p < 0.05 was considered statistically significant. Exact t-statistics, F-statistics, degrees of freedom, and post hoc p values are reported in the text SD: standard deviation; ANOVA: analysis of variance

Demographic characteristic	n	Knowledge score (mean ± SD)	Test statistic	p value	Attitude score (mean ± SD)	Test statistic	p value
Gender							
Male	444	6.60 ± 3.88	t(822) = -8.59^*^	<0.001	29.8 ± 4.79	t(868) = -5.83^*^	<0.001
Female	715	8.50 ± 3.26	t(822) = -8.59^*^	< 0.001	31.4 ± 4.33	t(868) = -5.83^*^	<0.001
Vaccination status							
Yes	134	8.35 ± 3.37	t(1,157) = -1.96^†^	0.050	30.8 ± 5.46	t(157) = 0.04^*^	0.972
No	1,025	7.70 ± 3.66	t(1,157) = -1.96^†^	0.050	30.8 ± 4.45	t(157) = 0.04^*^	0.972
Phase of study							
Phase I	503	6.13 ± 3.85	F(4, 284) = 65.0^‡^	<0.001	29.6 ± 4.50	F(4, 261) = 27.7^‡^	<0.001
Phase II	219	8.30 ± 3.42	F(4, 284) = 65.0^‡^	<0.001	30.4 ± 4.52	F(4, 261) = 27.7^‡^	<0.001
Phase III Part I	177	8.89 ± 2.44	F(4, 284) = 65.0^‡^	<0.001	31.9 ± 4.06	F(4, 261) = 27.7^‡^	<0.001
Phase III Part II	212	9.67 ± 2.56	F(4, 284) = 65.0^‡^	<0.001	32.9 ± 4.05	F(4, 261) = 27.7^‡^	<0.001
Internship	48	10.04 ± 2.15	F(4, 284) = 65.0^‡^	<0.001	32.3 ± 5.12	F(4, 261) = 27.7^‡^	<0.001
Residence							
Rural	515	7.82 ± 3.61	t(1,157) = 0.43^†^	0.669	30.5 ± 4.58	t(1,157) = -1.97^†^	0.049
Urban	644	7.73 ± 3.65	t(1,157) = 0.43^†^	0.669	31.0 ± 4.57	t(1,157) = -1.97^†^	0.049

Vaccinated students had a slightly higher mean knowledge score than unvaccinated students (8.35 ± 3.37 vs. 7.70 ± 3.66; Student’s t-test, t(1157) = −1.96, p = 0.050), though this did not reach the threshold for statistical significance (p = 0.050). Mean attitude scores were identical among vaccinated and unvaccinated students (30.8 in both groups; Welch’s t-test, t(157) = 0.04, p = 0.972). Knowledge scores did not differ significantly between rural and urban participants (7.82 ± 3.61 vs. 7.73 ± 3.65; Student’s t-test, t(1157) = 0.43, p = 0.669). However, urban participants had higher attitude scores than rural participants (31.0 ± 4.57 vs. 30.5 ± 4.58), and the difference was statistically significant (Student’s t-test, t(1157) = -1.97, p = 0.049).

Practice-related behaviors and preferences regarding HPV vaccination are presented in Table 6. Although only 134 (11.6%) participants had received the HPV vaccine, 461 (39.8%) had recommended HPV vaccination to another person. Participation in HPV awareness programs and patient counseling during clinical rotations was uncommon, reported by 171 (14.8%) and 119 (10.3%) participants, respectively. Nearly half, 565 (48.7%), expressed willingness to participate in outreach programs promoting HPV vaccination. Formal HPV-related training or workshops were considered necessary by 800 (69.0%), and 738 (63.7%) preferred interactive learning methods, such as case discussions and role-plays, over conventional lectures. Participants showed strong support for government provision of HPV vaccination free of cost, with a mean score of 8.56 ± 2.20 and a median of 10 (IQR: 8-10) on a 1-10 scale.

**Table 6:** Practice-related behaviors and educational preferences (n = 1,159) Categorical responses are presented as frequency (n) and percentage (%). Response categories are listed only when they were available for the corresponding item. For the clinical-counseling item, “not applicable” was available to students who had not commenced clinical postings. “Maybe” was available for the final three preference-related items. This table contains descriptive statistics only; no inferential statistical test was applied HPV: human papillomavirus

Practice item	Response	Value, n (%)
Vaccinated	Yes	134 (11.6)
	No	1,025 (88.4)
Recommended HPV vaccination to someone	Yes	461 (39.8)
	No	698 (60.2)
Participated in HPV awareness programs	Yes	171 (14.8)
	No	988 (85.2)
Conducted patient counseling regarding HPV vaccination during clinical rotations	Yes	119 (10.3)
	No	833 (71.9)
	Not applicable	207 (17.9)
Willing to participate in outreach programs promoting HPV vaccination	Yes	565 (48.7)
	No	235 (20.3)
	Maybe	359 (31.0)
Felt the need for formal training/workshops on HPV-related topics	Yes	800 (69.0)
	No	97 (8.4)
	Maybe	262 (22.6)
Preferred interactive learning methods (case discussions/role plays) over lectures	Yes	738 (63.7)
	No	106 (9.1)
	Maybe	315 (27.2)

The medical curriculum was the most frequently reported source of information regarding HPV, selected by 724/1,159 (62.5%) participants, followed by the internet, selected by 543/1,159 (46.9%), and healthcare professionals, selected by 474/1,159 (40.9%), as shown in Table 7. Media, seminars or workshops, and other sources were reported less frequently, whereas 80/1,159 (6.9%) participants reported having no source of HPV-related information. Among the reported reasons for nonvaccination, absence of a recommendation and lack of awareness were the most frequent. Other commonly reported reasons included not perceiving a need for vaccination, cost, and fear of adverse effects.

**Table 7:** Sources of information and reasons for nonvaccination Multiple responses were permitted for sources of information and reasons for nonvaccination; therefore, frequencies and percentages do not total 100%. Percentages for sources of information were calculated using all participants (n = 1,159). The reasons-for-nonvaccination item was optional. Of 1,025 unvaccinated participants, 1,009 selected a reason, and 16 did not provide a response. Percentages were calculated using all unvaccinated participants (n = 1,025) as the denominator

Domain	Response	Value, n (%)
Source of information	Medical curriculum	724 (62.5)
	Internet	543 (46.9)
	Healthcare professionals	474 (40.9)
	Media	301 (26.0)
	Seminars/workshops	281 (24.2)
	Other sources (peers, family, self-study)	274 (23.6)
	None (no source of information)	80 (6.9)
Reason for nonvaccination	Absence of recommendation	288 (28.1)
	Lack of awareness	287 (28.0)
	Did not feel the need	196 (19.1)
	Cost	163 (15.9)
	Fear of side effects	72 (7.0)
	Other reasons	3 (0.3)
	No response/no reason selected	16 (1.6)

Most participants did not believe that HPV vaccination was restricted to sexually active individuals, 933 (80.5%), or that vaccination against a sexually transmitted infection encouraged promiscuity, 718 (61.9%). Regarding peer-level discussion, 221 participants (19.1%) considered discussing HPV taboo among their peers, while 670 (57.8%) did not and 268 (23.1%) were uncertain, as shown in Table 8. However, gender was significantly associated with the belief that HPV vaccination encourages promiscuity, χ²(2, n = 1,159) = 35.8, p < 0.001, Cramer’s V = 0.176, indicating a small association. Male individuals were more likely than female individuals to answer “yes,” as answered by 67/444 (15.1%) vs. 70/715 (9.8%) female individuals. Conversely, female individuals were more likely to answer “no,” 491/715 (68.7%) vs. 227/444 (51.1%) male individuals. Nearly two-thirds, 778 (67.1%), believed that their parents would accept HPV vaccination for them. Nevertheless, 558 (48.1%) believed that vaccine-related myths deterred HPV vaccine uptake, whereas 351 (30.3%) were uncertain. Potential social judgment for receiving the vaccine was reported by 144 (12.4%), and 219 (18.9%) stated that they would feel uncomfortable discussing HPV-related topics with older family members.

**Table 8:** Social beliefs and perceived barriers Responses are presented as frequency (n) and percentage (%). The total sample size was 1,159. Selected associations with gender and HPV vaccination status were assessed using Pearson’s chi-square test. Cramer’s V was reported as a measure of effect size. All tests were two-sided, and p < 0.05 was considered statistically significant. Chi-square statistics, degrees of freedom, p values, and Cramer’s V values are reported in the text HPV: human papillomavirus

Statement	Yes, n (%)	No, n (%)	Maybe, n (%)
Do you think you'll be judged by society for wanting to receive HPV vaccination?	144 (12.4)	695 (60.0)	320 (27.6)
Do you think HPV vaccination is only for sexually active individuals?	52 (4.5)	933 (80.5)	174 (15.0)
Would you feel uncomfortable discussing HPV-related topics with older family members?	219 (18.9)	653 (56.3)	287 (24.8)
Do you believe vaccination for a sexually transmitted infection like HPV encourages promiscuity?	137 (11.8)	718 (61.9)	304 (26.2)
Would your parents accept HPV vaccination for you?	778 (67.1)	81 (7.0)	300 (25.9)
Would you personally face stigma if you publicly advocated for HPV vaccination?	102 (8.8)	695 (60.0)	362 (31.2)
Do you think myths about vaccines deter HPV vaccine uptake?	558 (48.1)	250 (21.6)	351 (30.3)
Are there religious objections to HPV vaccination in your community?	110 (9.5)	582 (50.2)	467 (40.3)
Do you think discussing HPV, a sexually transmitted infection, is taboo among your peers?	221 (19.1)	670 (57.8)	268 (23.1)

Overall, 686 (59.2%) participants agreed or strongly agreed that cultural beliefs influenced acceptance of HPV vaccination, as seen in Table 9. Religion was perceived to influence vaccination-related decision-making by 479 (41.3%), while 475 (41.0%) agreed that regional cultural norms discouraged open discussion of sexual health. The belief that the HPV vaccine might cause infertility because of inadequate research was endorsed by 140 (12.1%) participants, whereas 507 (43.7%) were neutral and 512 (44.2%) disagreed or strongly disagreed. Gender was significantly associated with responses to the statement that the HPV vaccine may cause infertility, χ²(4, n = 1,159) = 18.0, p = 0.001, Cramer’s V = 0.125, indicating a small association. Male individuals were more likely than female individuals to select “neutral” (213/444, 48.0% vs. 294/715, 41.1%) or “strongly agree” (21/444, 4.7% vs. 15/715, 2.1%). Female individuals were more likely to select “disagree” (234/715, 32.7% vs. 102/444, 23.0%). Vaccination status showed only small associations with social factors. The strongest associations were with believing that HPV vaccination is only for sexually active individuals, χ²(2) = 35.7, p < 0.001, Cramer’s V = 0.175, and that it encourages promiscuity, χ²(2) = 16.1, p < 0.001, Cramer’s V = 0.118. Parental acceptance was also significantly associated with vaccination status, χ²(2) = 11.0, p = 0.004. All other social factors were either weakly associated or not statistically significant.

**Table 9:** Cultural and religious perceptions and vaccine-related misconceptions Responses are presented as frequency (n) and percentage (%). The total sample size was 1,159. Selected associations of cultural, religious, and vaccine-related perceptions with gender and HPV vaccination status were assessed using Pearson’s chi-square test. Cramer’s V was reported as a measure of effect size. All tests were two-sided, and p < 0.05 was considered statistically significant. Chi-square statistics, degrees of freedom, p values, and Cramer’s V values are reported in the text HPV: human papillomavirus

Statement	Strongly disagree, n (%)	Disagree, n (%)	Neutral, n (%)	Agree, n (%)	Strongly agree, n (%)
Religion plays a role in decision-making regarding HPV vaccination	87 (7.5)	158 (13.6)	435 (37.5)	366 (31.6)	113 (9.7)
Cultural beliefs influence acceptance of HPV vaccination	61 (5.3)	92 (7.9)	320 (27.6)	479 (41.3)	207 (17.9)
Cultural norms in your region discourage open discussion of sexual health	77 (6.6)	188 (16.2)	419 (36.2)	359 (31.0)	116 (10.0)
The HPV vaccine may cause infertility due to inadequate research	176 (15.2)	336 (29.0)	507 (43.7)	104 (9.0)	36 (3.1)

A multivariable binary logistic regression model was fitted to identify factors independently associated with HPV vaccination uptake, as shown in Table 10. The overall model was statistically significant, χ²(8) = 34.2, p < 0.001, with a Nagelkerke R² of 0.057. Higher knowledge scores, female gender, and urban residence were independently associated with vaccination uptake. Each one-point increase in knowledge score was associated with 7.3% higher odds of having received the vaccine (aOR: 1.073, 95% CI: 1.009-1.141; p = 0.023). Female students had higher odds of vaccination than male students (aOR: 1.748, 95% CI: 1.149-2.658; p = 0.007), while urban students had higher odds than rural students (aOR: 2.212, 95% CI: 1.488-3.290; p < 0.001). Attitude score was not independently associated with vaccination status (aOR: 0.968 per one-point increase; 95% CI: 0.926-1.011; p = 0.148). The overall effect of phase of study was not statistically significant according to the omnibus likelihood-ratio test, χ²(4) = 8.16, p = 0.086. Although Phase II students had lower adjusted odds of vaccination than Phase I students in the individual category-specific comparison (aOR: 0.495, 95% CI: 0.280-0.876; p = 0.016), the comparisons for Phase III Part I, Phase III Part II, and internship were not statistically significant.

**Table 10:** Multivariable binary logistic regression analysis of factors associated with HPV vaccination uptake among medical students (n = 1,159) Multivariable binary logistic regression was performed to identify factors independently associated with HPV vaccination uptake among medical students (n = 1,159). The dependent variable was receipt of the HPV vaccine, coded as yes vs. no. All predictors were entered simultaneously into the model. Results are presented as aORs, 95% CIs, test statistics, and p values. Knowledge and attitude scores were entered as continuous variables. Male gender, Phase I, and rural residence were used as the reference categories. Likelihood-ratio chi-square statistics are reported for the overall effects of predictors, whereas Wald z-statistics are reported for individual phase-of-study comparisons. The overall model was statistically significant, likelihood-ratio χ²(8) = 34.2, p < 0.001, with a Nagelkerke R² of 0.057. All tests were two-sided, and p < 0.05 was considered statistically significant ^*^Statistically significant results aORs: adjusted odds ratios; CI: confidence interval; LR: logistic regression; HPV: human papillomavirus

Predictor	Comparison/reference category	Adjusted odds ratio	95% Confidence interval	Test statistic	p value
Knowledge score	Per one-point increase	1.073	1.009-1.141	LR χ²(1) = 5.16	0.023^*^
Attitude score	Per one-point increase	0.968	0.926-1.011	LR χ²(1) = 2.09	0.148
Gender	Female vs. male	1.748	1.149-2.658	LR χ²(1) = 7.19	0.007^*^
Phase of study, overall	Phase I as reference	-	-	LR χ²(4) = 8.16	0.086
Phase of study	Phase II vs. Phase I	0.495	0.280-0.876	z = -2.413	0.016^*^
	Phase III Part I vs. Phase I	0.758	0.435-1.321	z = -0.978	0.328
	Phase III Part II vs. Phase I	0.873	0.520-1.467	z = -0.513	0.608
	Internship vs. Phase I	0.425	0.143-1.263	z = −1.540	0.124
Place of residence	Urban vs. rural	2.212	1.488-3.290	LR χ²(1) = 16.47	< 0.001^*^

## Discussion

This multicenter study showed high prior awareness of HPV but uneven knowledge among MBBS students in Himachal Pradesh. Although 1,078 (93.0%) had heard of HPV, the mean knowledge score was 7.77 out of 15; 483 (41.7%) had lower knowledge, and only 181 (15.6%) had higher knowledge. Attitudes were more favorable, with a mean score of 30.8 out of 40 and 853 (73.6%) classified as favorable. However, only 134 (11.6%) had received an HPV vaccine. Most participants would recommend vaccination to family members and supported free vaccination for medical students. The largest knowledge deficits were regarding the full spectrum of HPV-associated diseases, the conditions for which vaccination provided protection, contraindications, and age-specific dose schedules. Knowledge and attitude scores were moderately correlated. In the adjusted model, higher knowledge, female gender, and urban residence were independently associated with vaccination uptake, whereas attitude score and study phase were not significant overall predictors.

Broad awareness was stronger than understanding of HPV infection and its vaccine. Most students correctly identified sexual transmission, the oncogenic role of high-risk HPV types, and the existence of multiple HPV types. Shetty et al. reported 89.3% HPV awareness and 78.0% knowledge of sexual transmission, whereas Sharma et al. found that 89.0% knew HPV was sexually transmitted and 86.9% linked HPV with cervical cancer [13,18]. Patel et al., who included female medical, nursing, and physiotherapy students at a rural university, reported much lower knowledge levels of the causation of cervical cancer [19]. Direct comparison of total scores is inappropriate because the studies used different questionnaires, score ranges, and cutoffs. In the present study, complete-response scoring was used for multiple-response items. This may partly explain why only 237 (20.4%) identified all HPV-associated diseases and 62 (5.3%) identified the full range of conditions prevented by vaccination, despite better performance on individual concepts.

Practical vaccine knowledge remained limited. Only 673 (58.1%) knew the ideal vaccination age, and 728 participants (62.8%) knew that male individuals should also receive HPV vaccination. Only 332 (28.6%) correctly identified contraindications, and 461 (39.8%) correctly answered the national immunization schedule item. This may reflect limited dissemination of information regarding the recent introduction of HPV vaccination into the national program [8].

Shetty et al. reported 38.3% correct knowledge of vaccination age, 41.0% knowledge of the dose requirement, 64.4% support for vaccination of both sexes, and 61.0% awareness that screening remains necessary after vaccination [13]. Patel et al. reported lower knowledge of age, doses, and vaccination of boys, at 14.0%, 14.0%, and 18.5%, respectively [19]. Knowledge that HPV vaccination may still be administered after prior HPV infection was limited, reported by only 22.9% of participants in Patel et al. and 31.98% in Singh and Baliga [19,20]. Omar et al. found that 60.2% knew both sexes should be vaccinated and 68.9% recognized noncervical HPV-related cancers [11].

Attitudes were generally favorable, but these outcomes were not directly comparable. In our study, 853 (73.6%) had a favorable attitude score. Support was high for vaccination of adolescent girls, free vaccination for medical students, recommendation to family members, and cervical cancer prevention. Support for vaccination of boys and men was lower, at 753 (65.0%). Concern about adverse effects was present in 394 (34.0%), whereas 500 (43.1%) were neutral. Sharma et al. [18] found that approximately 88% would accept vaccination if free; Patel et al. [19] reported 73.1% willingness; Singh and Baliga [20] reported 85.55% general willingness. Shetty et al. [13] reported 65.2% free-vaccine acceptance and 68.3% free-vaccine recommendation, whereas Omar et al. [11] reported 42.6% willingness among unvaccinated students, 58.9% acceptance if free, and 57.9% recommendation. The present results did not directly measure personal future vaccination intention.

Actual self-reported vaccination uptake remained low relative to awareness and favorable attitudes. Vaccination uptake, reported by 134/1,159 (11.6%) participants, was similar to that reported in other studies [11,20]. Patel et al. [19] reported 8.0% uptake. Sharma et al. [18] found vaccination in 15 of 143 female students and none of 95 male students, while Rehman et al. [21] reported 0.6% uptake among community women, and Sajan et al. [22] reported no vaccinated participants. Pal et al. estimated 4% community coverage, but excluded health-profession students and showed extreme heterogeneity [7]. In the present study, 461 (39.8%) had recommended vaccination to someone, yet only 134 participants (11.6%) were vaccinated. Lack of recommendation, lack of awareness, low perceived need, cost, and fear of adverse effects were reported. Intervention reviews found much higher uptake when education was combined with active vaccine delivery [9,23].

Several subgroup findings were notable in our study. Female students had higher knowledge and attitude scores and were more frequently vaccinated than male students, consistent with several previous studies [18,24]. Omar et al. [11] reported higher uptake among male individuals, and some studies, including Shetty et al. [13], found no sex difference in adequate knowledge or overall attitude. Knowledge increased across academic years, and Phase I students scored lower than all later groups. Similar patterns were reported by Sharma et al. [18] and Singh and Baliga [20], whereas Patel et al. [19] found no course-related difference. Urban and rural students had similar knowledge scores, but urban students had slightly higher attitude scores and a higher proportion reporting vaccination. Residence-related differences were also reported by Patel et al. [19], Rehman et al. [21], and Hussain et al. [24], although their populations and outcomes differed. In the adjusted model, knowledge remained associated with vaccination (aOR: 1.073 per point, 95% CI: 1.009-1.141; p = 0.023), as did female gender (aOR: 1.748, 95% CI: 1.149-2.658; p = 0.007) and urban residence (aOR: 2.212, 95% CI: 1.488-3.290; p < 0.001). Attitude score was not independently associated with vaccination uptake. The overall association between phase of study and vaccination uptake was not statistically significant (omnibus likelihood-ratio test, p = 0.086). Although Phase II students had lower adjusted odds of vaccination than Phase I students, this category-specific finding should be interpreted cautiously because the overall effect of phase of study was not statistically significant.

The medical curriculum was the leading source of information, followed by the internet and healthcare professionals. This finding was similar to other studies in which formal medical education was the dominant source [11,18,19,20]. Among unvaccinated students, absence of a recommendation and lack of awareness were the most frequent reasons, followed by low perceived need, cost, and fear of adverse effects. Almost half believed that vaccine myths deterred uptake. Most rejected the beliefs that vaccination is only for sexually active people or encourages promiscuity, but uncertainty remained. Cultural beliefs were perceived to influence vaccine acceptance by 686 (59.2%) participants, religion was perceived to influence decision-making by 479 (41.3%), and regional cultural norms were perceived to discourage sexual-health discussions by 475 (41.0%). Parental acceptance was expected by 778 (67.1%) participants. Vaccination status was associated with beliefs about sexual activity, promiscuity, and parental acceptance, although the associations were small and do not establish causation. Similar barriers were reported by others [9,11,19,23]. Sain et al. also found greater willingness at lower vaccine prices and a preference for information from healthcare providers [25].

The findings support focused educational and service-level responses within medical colleges. HPV teaching should begin early and be reinforced during clinical training. It should cover target age, age-specific dosing, vaccination of males, eligibility after sexual activity or prior infection, contraindications, and continued screening after vaccination. The need for formal training was reported by 800 (69.0%) participants, and a preference for interactive methods was reported by 738 (63.7%); these findings support expert-led workshops, case discussions, and counseling exercises. Teaching should prepare students to address safety, fertility, sexual behavior, and stigma-related concerns. Education alone may not be sufficient. Strong support for free provision, low uptake, and reported cost and recommendation barriers favor institution-based counseling and active vaccine offering at an affordable or subsidized price. Indian intervention evidence has shown consistent short-term knowledge gains, while programs that provided vaccination achieved much higher coverage than education-only or observational settings [9]. These findings support further implementation studies without implying causality or broader policy effects from the present cross-sectional data.

Strengths and limitations

This study had several strengths. It included 1,159 MBBS students from seven medical colleges across Himachal Pradesh, covering six government institutions and one private institution. Different academic phases were represented. The questionnaire assessed knowledge, attitude, vaccination practice, information sources, social beliefs, barriers, and educational preferences. The analysis included item-level comparisons, a knowledge-attitude correlation, and adjusted logistic regression. Because the study was cross-sectional, it could not determine cause-and-effect relationships. Vaccination status and other responses were self-reported and were not verified against vaccination records. Online, voluntary participation may have produced selection and nonresponse bias. Participation was uneven across colleges and academic years; phase I students were overrepresented, and interns formed a small group. Socially desirable responses were possible. The questionnaire underwent expert review and pilot testing, but the pilot study size was small, external validation was not reported, and the attitude-domain reliability was acceptable. Complete-response scoring for multiple-response items may have classified partially informed students as incorrect. Numerous exploratory bivariate comparisons were performed without adjustment for multiple testing, increasing the possibility of chance findings. Institution-level clustering was not accounted for, and the regression explained limited variation in vaccination status. Unmeasured factors such as affordability, vaccine access, and prior medical recommendation may have influenced uptake. Generalizability beyond medical students in Himachal Pradesh is limited.

## Conclusions

MBBS students in Himachal Pradesh had high HPV awareness but only moderate and unevenly detailed knowledge. Important deficits remained in disease spectrum, vaccine protection, contraindications, dosing, and gender-neutral vaccination. Attitudes were generally favorable, and substantially more students had recommended vaccination than had received it; nevertheless, uptake remained low. Higher knowledge, female gender, and urban residence were independently associated with uptake, whereas attitude score was not independently associated with vaccination. Medical colleges should strengthen practical, gender-neutral HPV education and pair counseling with easier and more affordable access to vaccination.

## Appendices

Study questionnaire

Section 1: Sociodemographic and Academic Characteristics and Prior Awareness of HPV (6 Items)

1. Age (years)

□ 17 or less

□ 18

□ 19

□ 20

□ 21

□ 22

□ 23

□ 24

□ 25

□ 26 or more

2. Gender

□ Male     □ Female

3. Year of study

□ Phase I / First Year (2025 batch)

□ Phase II / Second Year (2024 batch)

□ Phase III Part I / Third Year (2023 batch)

□ Phase III Part II / Final Year (2022 batch)

□ Internship (2021 batch)

4. Medical college

□ Indira Gandhi Medical College (IGMC), Shimla

□ Dr Rajendra Prasad Government Medical College (Dr RPGMC), Tanda

□ Shri Lal Bahadur Shastri Government Medical College (SLBSGMC), Mandi

□ Dr Yashwant Singh Parmar Government Medical College (Dr YSPGMC), Nahan

□ Dr Radhakrishnan Government Medical College (Dr RKGMC), Hamirpur

□ Pandit Jawaharlal Nehru Government Medical College (Pt JLNGMC), Chamba

□ Maharishi Markandeshwar Medical College (MMMC/MMU), Solan

5. Place of residence

□ Rural     □ Urban

6. Have you heard of Human Papillomavirus (HPV)?

□ Yes     □ No

Section 2: Knowledge Regarding HPV Infection and HPV Vaccination (15 Items)

1. HPV can cause which of the following? (Select all that apply)

□ Cervical cancer

□ Oropharyngeal cancer

□ Anal cancer

□ I don't know

2. What is the primary mode of HPV transmission?

□ Sexual contact

□ Blood transfusion

□ Vertical transmission (mother to child)

□ I don't know

3. Is cervical cancer caused only by HPV?

□ Yes     □ No     □ Maybe

4. Is there more than one type of HPV?

□ Yes     □ No     □ Maybe

5. High-risk HPV types are primarily associated with:

□ Cancer

□ Mild skin lesions

□ Respiratory infections

□ I don't know

6. At what age is HPV vaccination ideally recommended?

□ 9-14 years

□ 15-25 years

□ After sexual debut

□ I don't know

7. Does being vaccinated for HPV mean that a person cannot develop cervical cancer?

□ Yes     □ No     □ Maybe

8. Should men also receive HPV vaccination?

□ Yes     □ No     □ Maybe

9. How many doses are recommended for individuals aged 9-14 years?

□ 1

□ 2

□ 3

□ None

□ I don't know

10. How many doses are recommended for individuals older than 15 years?

□ Zero

□ 1

□ 2

□ 3

□ I don't know

11. Is the HPV vaccine included in India's National Immunization Schedule?

□ Yes     □ No     □ I don't know

12. Which of the following are contraindications to HPV vaccination? (Select all that apply)

□ Pregnancy

□ Severe allergy to a vaccine component

□ Immunocompromised state

□ I don't know

13. HPV vaccination provides protection against: (Select all that apply)

□ Cervical cancer

□ Penile cancer

□ All HPV-related cancers

□ I don't know

14. At what frequency should cervical cancer screening be performed in vaccinated women?

□ Every year

□ Every 3 years

□ Every 5 years

□ I don't know

15. Is routine cervical cancer screening still required after HPV vaccination?

□ Yes     □ No     □ Maybe

Section 3: Attitudes Towards HPV Infection and Vaccination (8 Items)

1. HPV is a serious infection.

□ Strongly agree

□ Agree

□ Neutral

□ Disagree

□ Strongly disagree

2. HPV vaccination should be mandatory for adolescent girls.

□ Strongly agree

□ Agree

□ Neutral

□ Disagree

□ Strongly disagree

3. HPV vaccination should be encouraged for boys/men as well.

□ Strongly agree

□ Agree

□ Neutral

□ Disagree

□ Strongly disagree

4. I worry about the side effects of the HPV vaccine.

□ Strongly agree

□ Agree

□ Neutral

□ Disagree

□ Strongly disagree

5. HPV vaccination should be provided free of cost to all medical students.

□ Strongly agree

□ Agree

□ Neutral

□ Disagree

□ Strongly disagree

6. I would recommend HPV vaccination to my family members.

□ Strongly agree

□ Agree

□ Neutral

□ Disagree

□ Strongly disagree

7. I feel confident explaining the benefits of HPV vaccination to society.

□ Strongly agree

□ Agree

□ Neutral

□ Disagree

□ Strongly disagree

8. I believe that HPV vaccination can reduce the incidence of cervical cancer in India.

□ Strongly agree

□ Agree

□ Neutral

□ Disagree

□ Strongly disagree

Section 4: Vaccination Practices, Vaccination-Related Behaviours and Educational Preferences (8 Items)

1. On a scale of 1-10, how supportive are you of the government providing HPV vaccination free of cost?

1 = Not supportive at all; 10 = Extremely supportive

□ 1     □ 2     □ 3     □ 4     □ 5     □ 6     □ 7     □ 8     □ 9     □ 10

2. Have you received the HPV vaccine?

□ Yes     □ No

3. Have you ever recommended HPV vaccination to someone?

□ Yes     □ No

4. Have you participated in any HPV awareness programmes?

□ Yes     □ No

5. Have you conducted any patient counselling about HPV vaccination during clinical rotations?

□ Yes     □ No     □ Not applicable

6. Would you participate in outreach programmes to promote HPV vaccination?

□ Yes     □ No     □ Maybe

7. Do you feel the need for more formal training or workshops on HPV-related topics?

□ Yes     □ No     □ Maybe

8. Would you prefer interactive sessions (case discussions and role-plays) over lectures for learning about HPV vaccination?

□ Yes     □ No     □ Maybe

Section 5: Social, Cultural, and Religious Beliefs and Misconceptions Regarding HPV and HPV Vaccination (13 Items)

1. Cultural beliefs influence the acceptance of HPV vaccination.

□ Strongly agree

□ Agree

□ Neutral

□ Disagree

□ Strongly disagree

2. Religion plays a role in decision-making regarding HPV vaccination.

□ Strongly agree

□ Agree

□ Neutral

□ Disagree

□ Strongly disagree

3. The HPV vaccine may cause infertility in the future due to lack of current research and testing.

□ Strongly agree

□ Agree

□ Neutral

□ Disagree

□ Strongly disagree

4. Do you think discussing HPV (a sexually transmitted infection) is taboo among your peers?

□ Yes     □ No     □ Maybe

5. Do you think you would be judged in society for wanting to receive HPV vaccination?

□ Yes     □ No     □ Maybe

6. Do you think HPV vaccination is only for sexually active individuals?

□ Yes     □ No     □ Maybe

7. Would you feel uncomfortable discussing HPV-related topics with older family members?

□ Yes     □ No     □ Maybe

8. Cultural norms in my region discourage open discussion of sexual health.

□ Strongly agree

□ Agree

□ Neutral

□ Disagree

□ Strongly disagree

9. Do you believe that a vaccine for a sexually transmitted infection such as HPV encourages promiscuity (having many transient sexual relationships)?

□ Yes     □ No     □ Maybe

10. Would your parents accept HPV vaccination for you?

□ Yes     □ No     □ Maybe

11. Would you personally face stigma if you publicly advocated for HPV vaccination?

□ Yes     □ No     □ Maybe

12. Do you think myths about vaccines deter HPV vaccine uptake?

□ Yes     □ No     □ Maybe

13. Are there religious objections to HPV vaccination in your community?

□ Yes     □ No     □ Not sure

Section 6: Sources of Information and Reasons for Non-vaccination (2 Items)

1. From where have you primarily obtained information about HPV and its vaccine? (Select all that apply)

□ Medical curriculum

□ Seminars/workshops

□ Internet

□ Peers

□ Healthcare professionals

□ Media

□ None

□ Other (please specify): ______________________________

2. If you have not received the HPV vaccine, what are the reasons? (Select all that apply)

Applicable only to participants who answered “No” to Section 4, Question 2.

□ Not aware

□ Did not feel the need

□ Cost

□ Absence of recommendation

□ Fear of side effects

□ Other (please specify): ______________________________
